# An updated view of glucose pathways in cyanobacteria

**DOI:** 10.7554/eLife.112405

**Published:** 2026-08-05

**Authors:** José Manuel García-Fernández

**Affiliations:** 1 Cyanotrans team, at the Departamento de Bioquímica y Biología Molecular, Campus de Excelencia Internacional Agroalimentario CeiA3, Universidad de Córdoba Córdoba Spain

**Keywords:** cyanobacteria, glucose metabolism, Entner-Doudoroff, glucose, enzymes, mixotrophy, Other

## Abstract

Contrary to previous belief, the cyanobacterium *Synechocystis* lacks the Entner-Doudoroff pathway for glucose metabolism.

**Related research article** Ojha RS, Theune M, Fritsche R, Makowka A, Boehm M, Peraglie C, Bräsen C, Snoep JL, Hagemann M, Siebers B, Gutekunst K. 2026. Story about honest mistakes: The cyanobacterium *Synechocystis* has a promiscuous Entner-Doudoroff (ED) aldolase but no functional ED pathway. *eLife* **15**:RP111485. doi: 10.7554/eLife.111485.

Cyanobacteria are photosynthetic microorganisms that can both produce glucose using solar energy (autotrophy) and import glucose or other organic compounds from their surroundings (mixotrophy/heterotrophy) (Cohen and Gurevitz, 2006; Stebegg et al., 2023; Muñoz-Marín et al., 2024). In cyanobacteria, glucose is then metabolized through central carbon metabolic pathways shared with other bacteria, such as classical glycolysis (also known as the Embden-Meyerhoff-Parnass pathway, EMP) or the oxidative pentose pathway, OPP (Figure 1A; Flamholz et al., 2013; Lucius and Hagemann, 2024).

**Figure 1.**
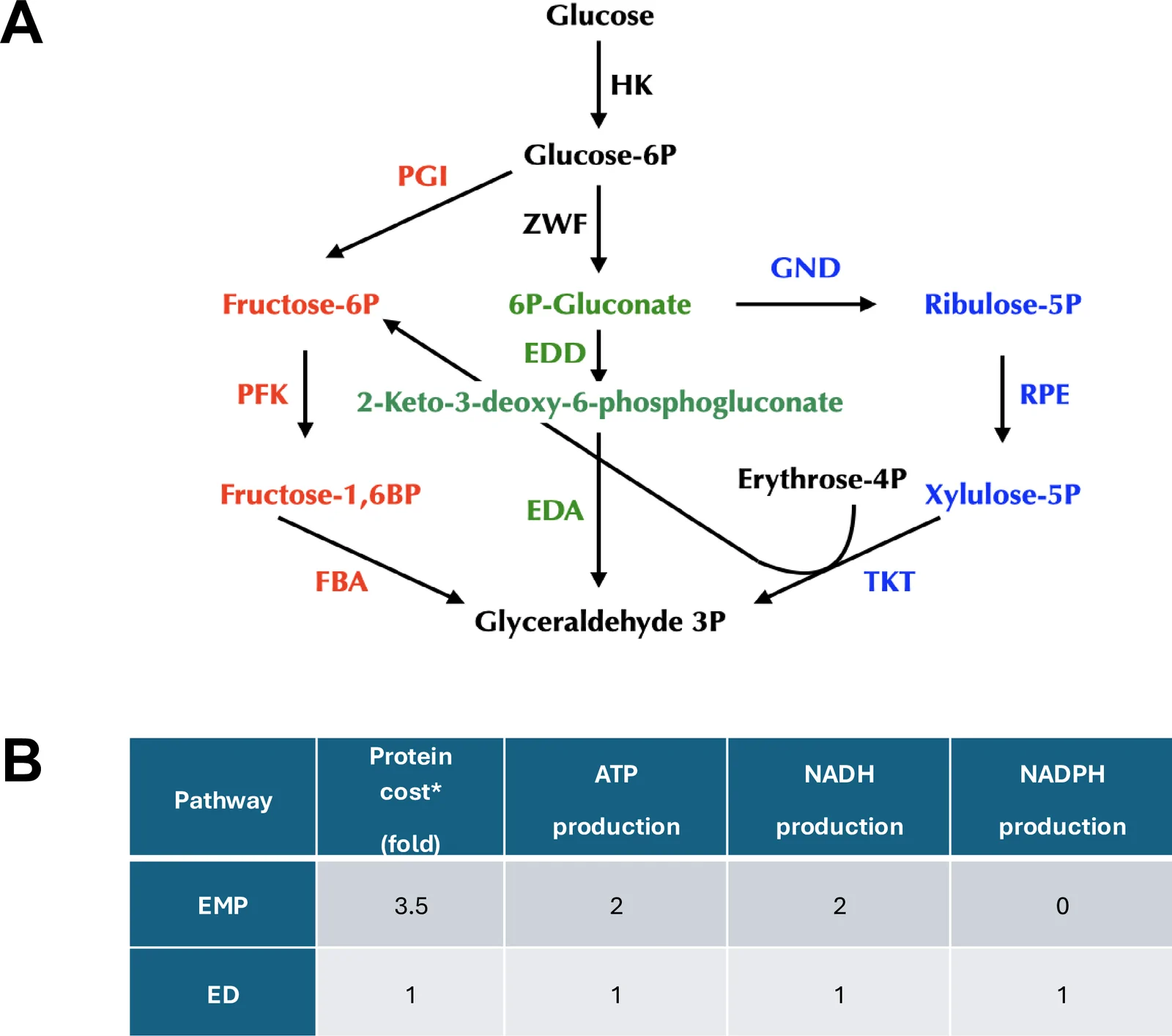
Glucose metabolite pathways in bacteria. (**A**) Simplified overview of the glucose degradation pathways found in cyanobacteria: the Embden–Meyerhof–Parnas (EMP, red), Entner–Doudoroff (ED, green), and oxidative pentose phosphate (OPP, blue) pathways and their shared intermediates. The figure shows only the reactions from glucose-6P to glyceraldehyde-3P, indicating the enzymes, substrates and products for each reaction. (**B**) Comparison of protein costs* vs energy production (per molecule of glucose) (ATP, NADH, NADPH) for the EMP and ED pathways. EDA, Entner-Doudoroff aldolase; EDD, Entner-Doudoroff dehydratase; FBA, fructose-1,6BP aldolase; GND, 6-phosphogluconate dehydrogenase; HK, hexokinase; KDPG, 2-keto-3-deoxy-6-phosphogluconate; PFK, phosphofructokinase; PGI, phosphoglucose isomerase; RPE, ribulose-5P epimerase; TKT, transketolase; ZWF, glucose-6-phosphate dehydrogenase. * According to Flamholz et al., 2013.

Previously, researchers proposed that some cyanobacteria can use a third route for degrading glucose, the Entner-Doudoroff (ED) pathway (Chen et al., 2016). Although this pathway produces less energy than the EMP (Figure 1B), the need for less enzymatic biomass would make it beneficial for cyanobacteria (Flamholz et al., 2013).

For photosynthetic microorganisms living under abundant light (i.e., not energy-limited) but subjected to nutrient restrictions, this might be a critical reason for favouring the ED pathway. Indeed, many cyanobacteria, especially marine strains such as *Prochlorococcus* and *Synechococcus,* live under such energetically strained conditions and lack key enzymes of the EMP pathway, including phosphofructokinase (Rocap et al., 2003).

However, later studies contradicted this proposal, indicating a lack of a true ED pathway in cyanobacteria: of the two enzymes exclusive to the ED pathway in *Synechocystis* – EDD and EDA – EDD appeared to be involved exclusively in amino acid synthesis, but not in glucose degradation, while EDA was shown to be a promiscuous, multifunctional enzyme using different substrates (Evans et al., 2024). Now, in eLife, Kirstin Gutekunst and colleagues – including Ravi Ojha and Marius Theune as joint first authors – report new insights into the glucose metabolite pathways of cyanobacteria (Ojha et al., 2026). The researchers, based at research institutes in Germany, the Netherlands and South Africa, used a combination of growth experiments, biochemical characterization and enzyme activity measurements in the cyanobacterium *Synechocystis* using wild-type and mutant strains.

Ojha et al. observed that the cyanobacterium lacked EDD. While *Synechocystis* has EDA, it is likely involved in the metabolism of phosphoenolpyruvate and proline, but not of glucose. This suggests that contrary to initial belief, the ED pathway does not actually exist in cyanobacteria.

In the current vision of the glucose utilization pathways in cyanobacteria proposed by Ojha et al., the main advantage of these pathways appears to be energy production rather than reducing the cost of enzyme biosynthesis. This suggests that, despite the potential evolutionary benefit of minimizing enzyme production under nutrient-limited conditions, particularly nitrogen limitation, maximizing energy generation is the predominant strategy (Figure 1B).

It is commendable that Ojha et al. clearly explained why previous studies reached incorrect conclusions and had the courage to thoroughly investigate the underlying causes of those errors, ultimately clarifying the metabolic landscape of glucose utilization in cyanobacteria. Furthermore, they openly discuss lessons learned from this experience, highlighting the importance of a careful biochemical characterization of enzymes when studying complex metabolic pathways; the risk of inferring gene function solely from sequence comparisons, or gene presence or absence; and the need to verify construct sequences after generating recombinant strains, as even minor sequence errors can profoundly affect experimental outcomes.

Future studies will help elucidate the actual physiological function of enzymes wrongly considered participants in the ED pathway in cyanobacteria, and to explore whether additional pathways for glucose utilization might exist in some species of cyanobacteria or plants.
